# Optimization of Surface Acoustic Wave Resonators on 42°Y-X LiTaO_3_/SiO_2_/Poly-Si/Si Substrate for Improved Performance and Transverse Mode Suppression

**DOI:** 10.3390/mi15010012

**Published:** 2023-12-21

**Authors:** Hongzhi Pan, Yang Yang, Lingqi Li, Qiaozhen Zhang, Zeyu Zheng, Xuesong Du, Pingjing Chen, Jiahe Dong, Chuan Lu, Xiao Xie, Hualin Li, Qiang Xiao, Jinyi Ma, Zhenglin Chen

**Affiliations:** 1 China Electronics Technology Group Corporation No.26 Research Institute (SIPAT), Chongqing 400060, China; plotinpan95@yeah.net (H.P.); 13752882051@163.com (Z.Z.); duxs@cetccq.com.cn (X.D.); 18202831317@163.com (P.C.); dongjh@cetccq.com.cn (J.D.); weeeee1984@163.com (C.L.); 62905695@163.com (X.X.); lihl@cetccq.com.cn (H.L.); xiaoqmail@foxmail.com (Q.X.); jymspe1970@163.com (J.M.); 2 College of Information, Mechanical and Electrical Engineering, Shanghai Normal University, Shanghai 200234, China; yyang@shnu.edu.cn (Y.Y.); 1000446645@smail.shnu.edu.cn (L.L.)

**Keywords:** SAW devices, comprehensive analysis, multi-layered structure, transverse mode suppression

## Abstract

SAW devices with a multi-layered piezoelectric substrate have excellent performance due to advantages such as a high quality factor, *Q*, low loss insertion, large bandwidth, etc. Prior to manufacturing, a comprehensive analysis and proper design are essential to evaluating the device’s key performance indicators, including the Bode *Q* value, bandwidth, and transverse mode suppression. This study explored the performance of SAW resonators employing a 42°Y-X LiTaO_3_ (LT) thin-plate-based multi-layered piezoelectric substrate. The thicknesses for each layer of the 42°Y-X LT/SiO_2_/poly-Si/Si substrate were optimized according to the index of phase velocity, Bode *Q* value, and bandwidth. The effect of the device structure parameters on the dispersion curve and slowness curve was studied, and a flat slowness curve was found to be favorable for transverse mode suppression. In addition, the design of the dummy configuration was also optimized for the suppression of spurious waves. Based on the optimized design, a one-port resonator on the 42°Y-X LT/SiO_2_/poly-Si/Si substrate was fabricated. The simulation results and measurements are presented and compared, which provides guidelines for the design of new types of SAW devices configured with complex structures.

## 1. Introduction

Surface acoustic wave (SAW) devices have been a key component in smart phones, cars, base stations, etc., due to their small size, good performance, and MEMS production process [1,2,3]. With the development of 5G communication technology, mobile communication has put forward a higher demand for the employed SAW filters, including a high frequency, large bandwidth, low loss, and good temperature stability.

The performance of SAW devices is mainly determined by their piezoelectric substrate. Traditional SAW devices, including the normal SAW structures and temperature-compensated SAW (TC-SAW) structures, are mainly based on a bulk piezoelectric single crystal [4,5]. Compared with those traditional ones, a piezoelectric thin film based on a multi-layered structure [3,6] offers not only a higher frequency and larger coupling factor (*K*^2^) but also a higher Bode *Q* value [7] and a moderate temperature coefficient of frequency (TCF). These excellent characteristics have attracted much attention for SAW devices with multi-layered structures, which have been widely used in RF filters in the consumer market [8,9,10,11].

Although SAW devices with multi-layered structures have a distinct advantage, the design of one with an optimized performance calls for intensive study. Prior to manufacturing, a comprehensive analysis and proper design are essential to evaluating its key performance indicators, including the Bode *Q* value, bandwidth, and transverse mode suppression. In a previous study, T. Takai et al. studied the *K*^2^ and TCF of SAWs on LiTaO_3_ (LT) thin-film-based multi-layered structures and successfully applied them to a high-performance SAW filter [8,12]. Recently, researchers have concentrated on the suppression of the transverse mode because it appears within the passband of the filter and thus affects the ripple and passband loss. There are some solutions for this thorny issue, for example, the use of a piston mode [13,14], apodization [15,16], and tilted IDT [9]. S. Inoue et al. [17] also pointed out that an LT/quartz-layered SAW substrate with a flat slowness curve can obtain good suppression of transverse modes. Those previous works suggest that LiTaO_3_ thin-film-based multi-layered structures are good candidate piezoelectric substrates for high-performance SAW devices. Meanwhile, the employed materials and thicknesses of the multi-layered substrate can modulate the flatness of the slowness curve, which makes transverse mode suppression possible. However, there is a need for a comprehensive analysis prior to manufacturing a desired SAW device.

Therefore, in this paper, a piezoelectric substrate with a 42°Y-X LT/SiO_2_/poly-Si/Si multi-layered structure is proposed. The analytical theory and finite element method were employed to comprehensively analyze the SAW resonator based on the proposed layered structure. First, we studied the constitutive relationship between the mechanical displacement and electric field in the piezoelectric thin film, and then the internal relationship between the slowness curve and dispersion characteristic of the SAWs between the propagation and aperture directions was derived. Second, the device performance, including the admittance characteristic, Bode Q value, and bandwidth, was calculated. The effect of the piezoelectric thin film and electrode thickness on the performance of the SAWs on the 42°Y–X LT/SiO_2_/poly-Si/Si substrate was studied, which can provide guidelines for the comprehensive design of SAW devices. Next, the dispersion characteristic was analyzed for the suppression of spurious transverse modes generated by the boundary effect of the electrode aperture. Then, the appropriate structure configurations of the substrate and electrode were obtained according to dispersion and slowness curves. Moreover, in order to ensure the suppression of spurious waves, a dummy structure based on the optimized design, a one-port resonator on the 42°Y-X LT/SiO_2_/poly-Si/Si substrate, was fabricated. The simulation results and measurements were compared.

## 2. Simulation Techniques

For piezoelectric devices, the constitutive relationship between the mechanical displacement and electric field can be described through the multi-physical coupling in Equations (1) and (2) [18,19]:

(1) $${Τ}_{I}=c_{IJ}S_{J}-e_{Ij}E_{j}$$

(2) $$D_{i}=e_{iJ}S_{J}+\varepsilon_{ij}E_{j}$$

where ${Τ}_{I}$ and $S_{J}$ are stress and strain tensors, respectively; $c_{IJ}$ and $\varepsilon_{ij}$ are the stiffness constant and dielectric permittivity constants; $e_{iJ}$ and $e_{Ij}$ are both piezoelectric stress constants; and $D_{i}$ and $E_{j}$ are the electric displacement vector and electric field, respectively.

The relationship between the strain and mechanical displacement can be described as

(3) $$S_{J}=\nabla_{iJ}u_{i}$$

where

(4) $$\nabla_{iJ}=\left[\begin{array}{cccccc} \frac{\partial }{\partial x_{1}} & 0 & 0 & 0 & \frac{\partial }{\partial x_{3}} & \frac{\partial }{\partial x_{2}}\\ 0 & \frac{\partial }{\partial x_{2}} & 0 & \frac{\partial }{\partial x_{3}} & 0 & \frac{\partial }{\partial x_{1}}\\ 0 & 0 & \frac{\partial }{\partial x_{3}} & \frac{\partial }{\partial x_{2}} & \frac{\partial }{\partial x_{1}} & 0 \end{array}\right]$$

**Figure 1:**
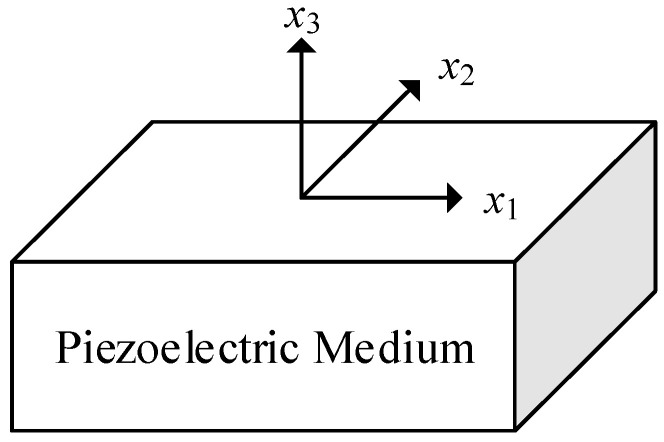
The coordinate system of SAW propagation in a piezoelectric material.

As shown in Figure 1, $x_{1}$ represents the direction of wave propagation, $x_{2}$ represents the direction of the aperture, and $x_{3}$ represents the direction of the substrate thickness.

According to Maxwell’s equations and the boundary conditions of SAW devices, the relationships between the electric displacement, $D_{i}$, electric field, $E_{j}$, electric potential, $\phi_{j},$ and charge density, $\rho_{s},$ are expressed as

(5) D=εE

(6) E=−∇ϕ

(7) $$\nabla \cdot D=\rho_{s}$$

where the Nabla operator is $\nabla =\left[\begin{array}{ccc} \frac{\partial }{\partial x_{1}} & \frac{\partial }{\partial x_{2}} & \frac{\partial }{\partial x_{3}} \end{array}\right]$. Equation (7) is applied to the interface between the piezoelectric thin film and electrodes. Additionally, $\rho_{s}=0$ is applied to the piezoelectric thin film.

Assuming that there is no external force applied, the equilibrium equation in the piezoelectric medium can be described in the tensor form:

(8) $$\nabla_{iJ}c_{JK}\nabla_{Kl}u_{l}+\nabla_{iJ}e_{JK}\nabla \phi =\rho {\ddot{u}}_{i}$$

(9) $$-\nabla_{i}\varepsilon_{im}\nabla_{m}\phi +\nabla e_{iK}\nabla_{Kj}u_{j}=0$$

In this work, the thorough consideration of SAW devices consists of two aspects: (1) The analysis of the substrate structure. We assume that the length of the aperture along the $x_{2}$ direction is infinite (*∂*/∂$x_{2}$= 0) to research the regulator of the SAW device structure and find the appropriate size according to the Bode *Q* values, the relative bandwidth, etc. Therefore, the operator $\nabla_{iJ}$ and Nabla operator ∇ can be expressed as the following equation:

(10) $$\nabla_{iJ}=\left[\begin{array}{cccccc} \frac{\partial }{\partial x_{1}} & 0 & 0 & 0 & \frac{\partial }{\partial x_{3}} & 0\\ 0 & 0 & 0 & \frac{\partial }{\partial x_{3}} & 0 & \frac{\partial }{\partial x_{1}}\\ 0 & 0 & \frac{\partial }{\partial x_{3}} & 0 & \frac{\partial }{\partial x_{1}} & 0 \end{array}\right]$$

(11) $$\nabla =\left[\begin{array}{ccc} \frac{\partial }{\partial x_{1}} & 0 & \frac{\partial }{\partial x_{3}} \end{array}\right]$$

In addition, many methods for spurious suppression are applied to improve the properties of SAW devices, but these kinds of methods generally have different effects on spurious suppression. (2) In the analysis of the spurious wave yield at the aperture boundary, it is assumed that the length of the substrate along the $x_{3}$ direction is infinite (∂/∂$x_{3}$ = 0). The operator $\nabla_{iJ}$ and Nabla operator ∇ can be written as

(12) $$\nabla_{iJ}=\left[\begin{array}{cccccc} \frac{\partial }{\partial x_{1}} & 0 & 0 & 0 & 0 & \frac{\partial }{\partial x_{2}}\\ 0 & \frac{\partial }{\partial x_{2}} & 0 & 0 & 0 & \frac{\partial }{\partial x_{1}}\\ 0 & 0 & 0 & \frac{\partial }{\partial x_{2}} & \frac{\partial }{\partial x_{1}} & 0 \end{array}\right]$$

(13) $$\nabla =\left[\begin{array}{ccc} \frac{\partial }{\partial x_{1}} & \frac{\partial }{\partial x_{2}} & 0 \end{array}\right]$$

Substituting Equations (10) and (11) into the equilibrium Equations (8) and (9), the wave propagation characteristic along the aperture can be described using the following equation:

(14) $$\left\{\begin{array}{c} c_{11}\frac{\partial^{2}u_{1}}{\partial x_{1}^{2}}+{(c}_{61}+c_{16})\frac{\partial^{2}u_{1}}{\partial x_{1}\partial x_{2}}+c_{66}\frac{\partial^{2}u_{1}}{\partial x_{2}^{2}}+{(c}_{12}+c_{66})\frac{\partial^{2}u_{2}}{\partial x_{1}\partial x_{2}}+c_{16}\frac{\partial^{2}u_{2}}{\partial x_{1}^{2}}+c_{62}\frac{\partial^{2}u_{2}}{\partial x_{2}^{2}}=\rho \frac{\partial^{2}u_{1}}{\partial t^{2}}\\ c_{61}\frac{\partial^{2}u_{1}}{\partial x_{1}^{2}}+{(c}_{21}+c_{66})\frac{\partial^{2}u_{1}}{\partial x_{1}\partial x_{2}}+c_{26}\frac{\partial^{2}u_{1}}{\partial x_{2}^{2}}+{(c}_{62}+c_{26})\frac{\partial^{2}u_{2}}{\partial x_{1}\partial x_{2}}+c_{66}\frac{\partial^{2}u_{2}}{\partial x_{1}^{2}}+c_{22}\frac{\partial^{2}u_{2}}{\partial x_{2}^{2}}=\rho \frac{\partial^{2}u_{1}}{\partial t^{2}} \end{array}\right.$$

As the piezoelectric material is an orthotropic material such as LiNbO_3_ and LiTaO_3_, Equation (14) can take a simplified form [20]:

(15) $$\left\{\begin{array}{c} c_{11}\frac{\partial^{2}u_{1}}{\partial x_{1}^{2}}+{(c}_{12}+c_{66})\frac{\partial^{2}u_{2}}{\partial x_{1}\partial x_{2}}+c_{66}\frac{\partial^{2}u_{1}}{\partial x_{2}^{2}}=\rho \frac{\partial^{2}u_{1}}{\partial t^{2}}\\ C_{66}\frac{\partial^{2}u_{2}}{\partial x_{1}^{2}}+{(c}_{21}+c_{66})\frac{\partial^{2}u_{1}}{\partial x_{1}\partial x_{2}}+c_{22}\frac{\partial^{2}u_{2}}{\partial x_{2}^{2}}=\rho \frac{\partial^{2}u_{2}}{\partial t^{2}} \end{array}\right.$$

The SAW propagates in the $x_{1}x_{2}$ plane, and a coupling phenomenon occurs. The equation of the particle displacement is described as

(16) $$u_{i}=A_{i}\exp\left[j\left(\omega t-k_{1}x_{1}-k_{2}x_{2}\right)\right]$$

Substituting Equation (16) into Equation (15), we obtain:

(17) $$\left\{\begin{array}{c} c_{11}\frac{\partial^{2}u_{1}}{\partial x_{1}^{2}}+{(c}_{12}+c_{66})\frac{\partial^{2}u_{2}}{\partial x_{1}\partial x_{2}}+c_{66}\frac{\partial^{2}u_{1}}{\partial x_{2}^{2}}=-\rho \omega^{2}u_{1}\\ C_{66}\frac{\partial^{2}u_{2}}{\partial x_{1}^{2}}+{(c}_{21}+c_{66})\frac{\partial^{2}u_{1}}{\partial x_{1}\partial x_{2}}+c_{22}\frac{\partial^{2}u_{2}}{\partial x_{2}^{2}}=-\rho \omega^{2}u_{2} \end{array}\right.$$

According to Equation (16), the wave number domain is described as

(18) $$\left\{\begin{array}{c} c_{11}\left(-k_{1}^{2}u_{1}\right)+c_{66}\left(-k_{2}^{2}u_{1}\right)+\rho \omega^{2}u_{1}+{(c}_{12}+c_{66})\left(-k_{1}k_{2}u_{2}\right)=0\\ c_{66}\left(-k_{1}^{2}u_{2}\right)+c_{22}\left(-k_{2}^{2}u_{2}\right)+\rho \omega^{2}u_{2}+{(c}_{21}+c_{66})\left(-k_{1}k_{2}u_{1}\right)=0 \end{array}\right.$$

According to the solutions to Equations (17) and (18), the frequency dispensation and wave number domain can be plotted and are applied to characterize the coupling of the wave propagation along the $x_{1}x_{2}$ plane.

## 3. Results and Discussion

### 3.1. Analysis of Piezoelectric Thin-Film-Based Multi-Layered Structure

**Figure 2:**
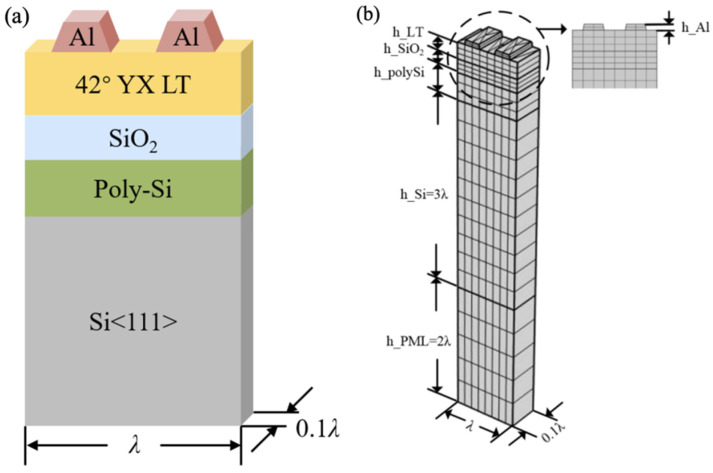
Periodic model of multi-layered structure. (**a**) Schematic diagram of structural materials; (**b**) mesh distribution of finite element model.

The high performance of SAWs on a piezoelectric thin-film-based multi-layered structure is due to the utilization of the advantages of various materials. The energy is confined to the surface due to the combination of low-velocity films and a high-velocity substrate. Here, we present a comprehensive analysis of a SAW resonator with a 42°Y-X LT substrate. As shown in Figure 2, it uses a SiO_2_ material as the temperature-compensated layer, a polysilicon (poly-Si) material as the trapping layer, and Si as the support substrate.

Without a loss of generality, the surface acoustic wave is assumed to propagate in the $x_{1}$ direction on a multi-layered structure. In order to simplify the solution while maintaining a good enough accuracy, the full-scaled 3D finite element model (FEM) was decomposed into a double-finger structure with one period interdigital transducer (IDT) [21,22,23,24,25]. In addition, a substrate with a perfect matching layer set to the bottom for absorbing the wave propagated into the substrate was constructed. The continuity periodic boundary condition was set on the side of the model to extend it in the X direction to infinity. Additionally, it is seen that the geometric shape of the electrode is trapezoid because of the practical processing. And the mesh size of the region below the electrode was smaller than the other regions of the substrate due to the energy of the acoustic surface wave mainly focusing on the surface of the piezoelectric thin film. The maximum element size of the electrode was λ/6. The material constants used in the calculation are listed in Table 1.

**Figure 3:**
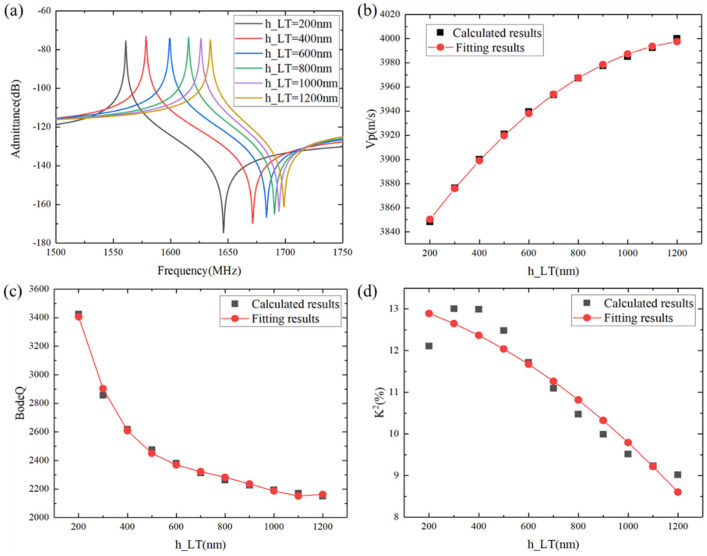
Calculated characteristic changes of the SH wave with different LiTaO_3_ thicknesses. (**a**) Admittance, (**b**) *V*_p_, (**c**) Bode *Q*, and (**d**) *K*^2^. Black shapes denote the calculated data, and red shapes denote the fitting results.

To attain excellent performance in this layer structure, the thicknesses of the different layers were optimized through frequency domain simulations by the MUMPS solver using the infinite periodic models shown in Figure 2. The changes in the frequency characteristic with respect to the LiTaO_3_ thickness are shown in Figure 3. In this case, the IDT period, λ, was 2.4 µm, the metallization ratio of the IDT was 0.5, the Al electrode thickness was 170 nm, the SiO_2_ thickness was 500 nm, and the poly-Si thickness was fixed to 1 µm. In the following calculation, the thickness of the bottom Si substrates was set as 3λ, and the thickness of the PML was set as 2λ. Figure 3a presents a comparison of the calculated admittance Y_11_ curves of the SAW resonators with increasing LiTaO_3_ thickness. It is obvious that the LiTaO_3_ thickness has a great influence on the frequency characteristic, as the resonant frequency gradually decreased with the increase in the LiTaO_3_ thickness. In SAW applications, spurious waves are not allowed to exist. Figure 3b clearly shows the dependency of the phase velocity (*V_p_*) on LiTaO_3_ thickness, with the phase velocity increasing with increasing LiTaO_3_ thickness. This is due to the LiTaO_3_ thickness-induced dispersion of the phase velocity for high-frequency SAWs, where high-velocity acoustic waves are expected. Figure 3c shows the effect of the LiTaO_3_ thickness on Bode *Q* values; the Bode *Q* values decrease with increasing LiTaO_3_ thickness. For the low insertion of SAW devices, a special multi-substrate structure with high Bode *Q* is used. The electromechanical coupling coefficient (*K*^2^) is illustrated in Figure 3d, where it can be seen that the *K*^2^ value gradually decreased as the LiTaO_3_ thickness increased. For SAWs that require a large bandwidth, a specific LiTaO_3_ thickness with a large bandwidth is suitable for implementation. Based on the above simulation results, it can be seen that the maximum *Q* value of I.H.P. SAW resonators reached 3400, which is three times higher than that of the standard 42°Y-X LT SAW resonator; in addition, the maximum *K*^2^ value of I.H.P. SAW resonators exceeded 12%, which is a 20% wider bandwidth than that of normal SAWs [8,9].

With the increase in the LiTaO_3_ thickness, the resonance frequency, phase velocity, Bode *Q* value, and electromechanical coupling coefficient (*K*^2^) changed monotonously. This is because the energy of the SH wave is mainly concentrated on the device’s surface. When the piezoelectric thin film becomes thicker, its performance is similar to that of an SH wave on a 42°Y-X LT structure.

**Figure 4:**
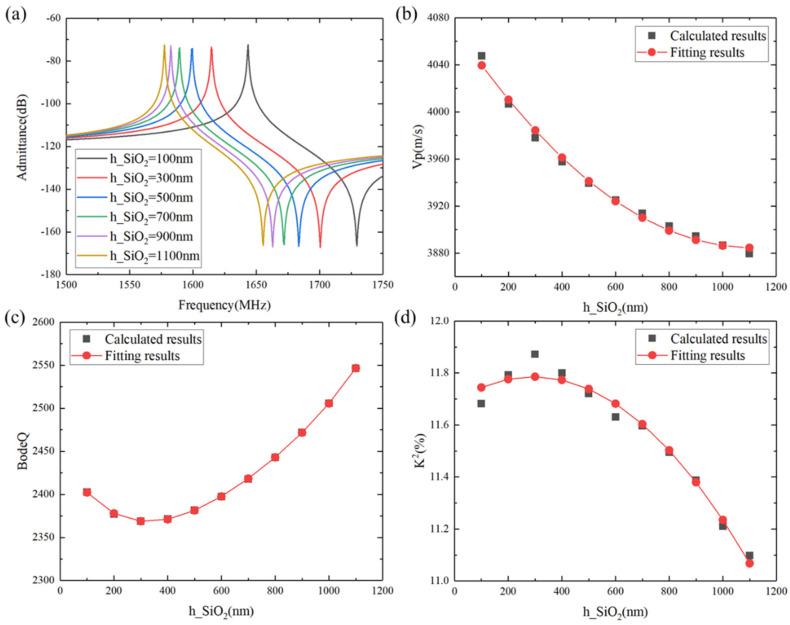
Calculated characteristic changes of the SH wave with SiO_2_ thickness. (**a**) Admittance, (**b**) *V*p, (**c**) Bode *Q*, and (**d**) *K*^2^. Black shapes denote the calculated data, and red shapes denote the fitting results.

Furthermore, the effect of the temperature-compensated SiO_2_ layer on the comprehensive performance also deserves attention. In this case, the IDT period λ = 2.4 µm, the metallization ratio of the IDT was 0.5, the Al electrode thickness was 170 nm, the LiTaO_3_ thickness was 600 nm, and the poly-Si thickness was fixed to 1 µm. Figure 4a illustrates the calculated admittance Y_11_ curves as the SiO_2_ thickness increases. The resonance frequency monotonously decreased with the increase in the SiO_2_ thickness. Figure 4b shows the phase velocity vs. SiO_2_ thickness curve; it can be clearly seen that the phase velocity decreased with increasing SiO_2_ thickness. Figure 4c shows the effect of the SiO_2_ thickness on the Bode *Q* values. It can be seen that the Bode *Q* values exhibited a nonlinear increase as a whole as the SiO_2_ thickness increased. Figure 4d illustrates the *K*^2^ vs. SiO_2_ thickness curve, where it is clear that the *K*^2^ value appears to decline in a non-linear manner with the increase in SiO_2_ thickness. The *K*^2^ value reached its maximum at around 300 nm SiO_2_.

**Figure 5:**
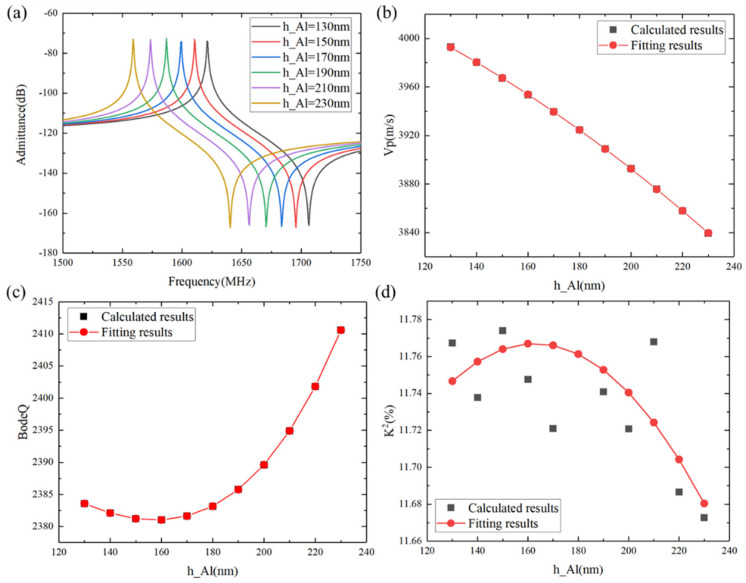
Calculated characteristic changes of the SH wave with different Al thicknesses. (**a**) Admittance, (**b**) *V*_p_, (**c**) Bode *Q*, and (**d**) *K*^2^. Black shapes denote the calculated data, and red shapes denote the fitting results.

In addition to the LiTaO_3_ and SiO_2_ thicknesses, the electrode thickness also affects SAW performance due to mass loading, as shown in Figure 5. In this case, the IDT period λ = 2.4 µm, the metallization ratio of the IDT was 0.5, the LiTaO_3_ thickness was 600 nm, the SiO_2_ thickness was 500 nm, and the poly-Si thickness was 1 µm. Figure 5a illustrates how the calculated admittance Y_11_ curve changes with increasing Al thickness. It can be clearly seen that the mass loading of the Al electrode led to a monotonous decrease in the resonant frequency as the Al thickness increased. Meanwhile, the wave modes in the piezoelectric medium have different excitation efficiencies, and therefore a suitable Al thickness was selected to suppress spurious waves within the passband range. The phase velocity, Bode *Q* values, and *K*^2^ values showed similar curves to those with increasing SiO_2_ thickness, as can be clearly seen in Figure 5b–d.

**Figure 6:**
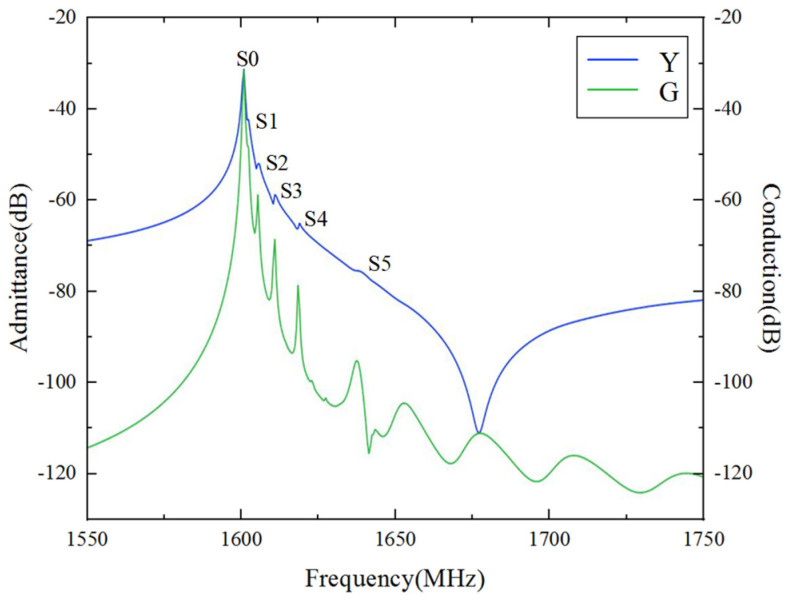
3D FEM simulation results.

**Figure 7:**
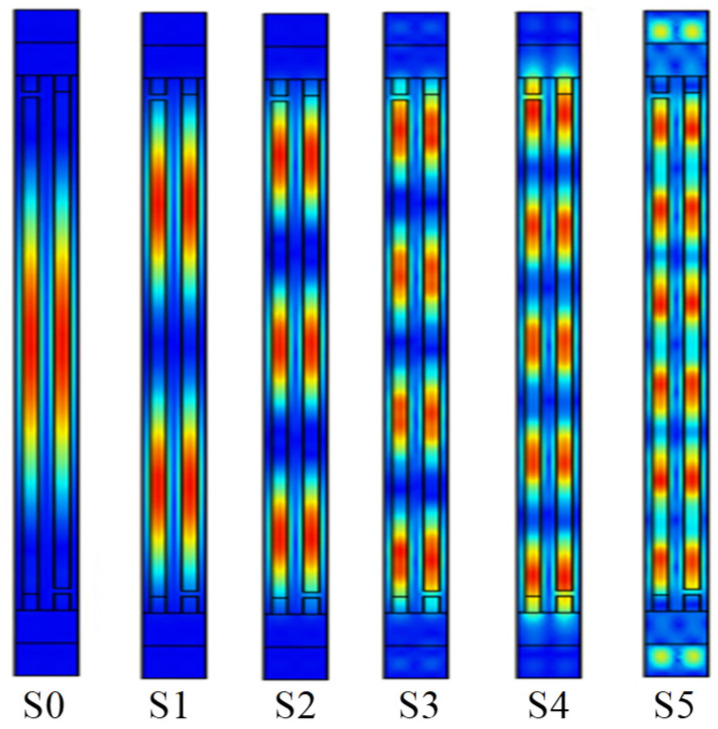
Displacement of higher-order transverse modes along the aperture direction.

According to the above analysis, we found that the thickness of LiTaO_3_ has a significant impact on the Bode *Q* and *K*^2^ values, while the thicknesses of SiO_2_ and Al mainly affect the speed. In practical applications, a local finite element simulation cannot fully characterize the performance of the model in the aperture direction. In order to maintain generality, we used Al (170 nm)/42°Y-X LT (600 nm)/SiO_2_ (500 nm)/ploy-Si/Si to build a 3D periodic model with a gap length of 0.175*λ*, a dummy length of 0.5*λ*, a dummy width of 0.25*λ*, and an aperture length of 15*λ*. The finite element mesh of the 3D periodic model is made of free tetrahedral elements. In this analysis, a degree of freedom (DOF) of about 203,761 could be obtained. As shown in Figure 6, the result exhibited multiple higher-order harmonics between the resonance frequency and the anti-resonance frequency. Figure 7 shows the displacement of each higher-order transverse mode (S1, S2, S3, S4, and S5) along the aperture direction. The surface wave propagates back and forth many times in its resonant cavity while undergoing total reflection at the busbars and reflectors, thus causing the SAW to propagate laterally. The lateral resonant energy exists as higher harmonics near the main resonant frequency. These responses would cause a large amount of ripple in the passband of the SAW filter, affecting the loss and flatness of the device.

### 3.2. Analysis of Spurious Suppression

**Figure 8:**
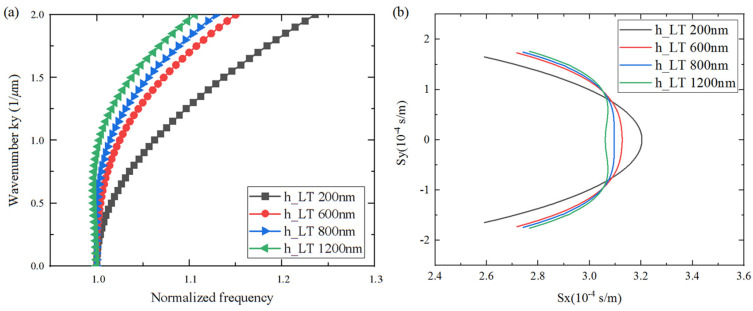
Calculated changes in dispersion/slowness curves for different LiTaO_3_ thicknesses. (**a**) Dispersion curves; (**b**) Slowness curves.

**Figure 9:**
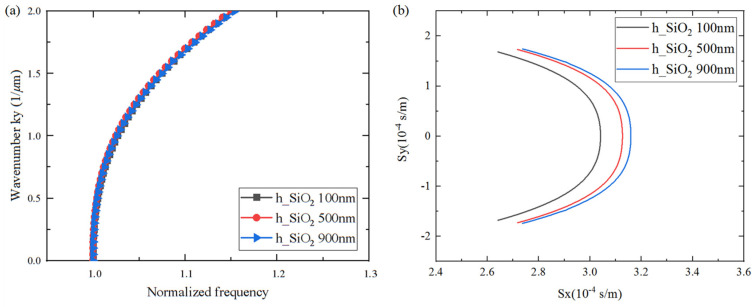
Calculated changes in dispersion/slowness curves for different SiO_2_ thicknesses. (**a**) Dispersion curves; (**b**) Slowness curves.

**Figure 10:**
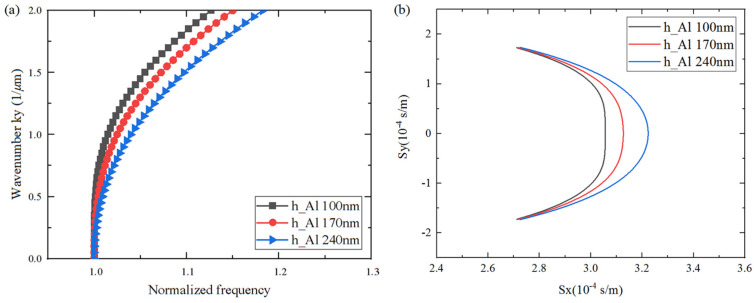
Calculated changes in dispersion/slowness curves for different Al thicknesses. (**a**) Dispersion curves; (**b**) Slowness curves.

The occurrence of transverse modes is mainly due to aperture boundary effects, which are associated with the variation in the slowness curve. In order to conduct a rigorous and comprehensive analysis, the influence of LiTaO_3_, SiO_2_, and Al thickness on the slowness curve shape was investigated for the transverse mode suppression on a Al/42°Y-X LT/SiO_2_/poly-Si/Si substrate. As shown in Figure 8, Figure 9 and Figure 10, the *x*-axis represents the normalized frequency and the slowness, *S_x_*, of the horizontal propagation direction, and the *y*-axis represents the wave number, *k_y_*, and *S_y_* along the aperture propagation direction. It can be seen that the dispersion and slowness curves show flat, convex, or concave shapes, i.e., the thickness of each layer has different effects on the curvature of the dispersion curve. When the LiTaO_3_ film thickness changed from 200 nm to 1200 nm, the shape of the dispersion curve changed from convex to concave. This is attributed to the increasing influence of the concave dispersion curve of the 42°Y-X LT. This conclusion is also consistent with the results in Figure 3. Nevertheless, the SiO_2_ thickness had a weak effect on the curvature of the dispersion curve, mainly on the wave mode velocity, manifested by a parallel shift of the slowness curve. Lastly, the Al thickness had a strong effect on the curvature of the dispersion curve. For the flat curve, the main wave mode formed a standing wave in the IDT region, with the direction of energy propagation only along the horizontal direction. A convex or concave curve means that the energy is a propagating component of the wave in the aperture direction. Therefore, a flat dispersion curve and slowness at a specific thickness were taken into consideration.

**Figure 11:**
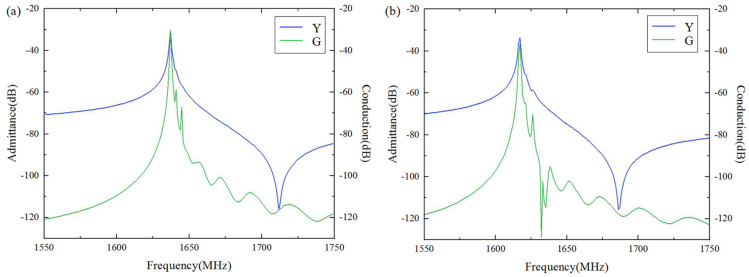
Three-dimensional FEM simulation results. (**a**) The Al electrode (100 nm)/42°Y-X LT (600 nm) structure; (**b**) The Al electrode (170 nm)/42°Y-X LT (800 nm)/Si structure.

Corresponding to the flat slowness curves in Figure 8 and Figure 10, the calculated frequency responses of the Al (100 nm)/42°Y-X LT (600 nm)/SiO_2_/poly-Si/Si and Al electrode (170 nm)/42°Y-X LT (800 nm)/SiO_2_/poly-Si/Si are shown in Figure 11. The poly-Si thickness was 1 µm. The calculated results show that the effects on the suppression of transverse modes of these two structures are reasonable. Although the lateral high-order wave existed near the main resonant frequency, its acoustic energy was still weak.

**Figure 12:**
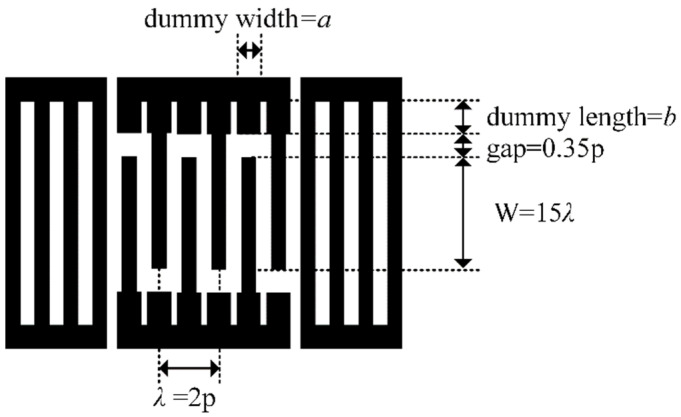
Electrode configuration of resonator.

During fabrication, there are manufacturing errors in the electrode and film size of practical SAW devices. For example, electrodes are usually trapezoid in shape due to the actual process. These errors probably lead to a change in the flat slowness curve. In order to ensure good production yield and performance, the dummy structure for assisting in improving SAW performance was also described, as shown in Figure 12, with a LiTaO_3_ thickness of 600 nm, a SiO_2_ thickness of 500 nm, and an Al electrode thickness of 170 nm.

**Figure 13:**
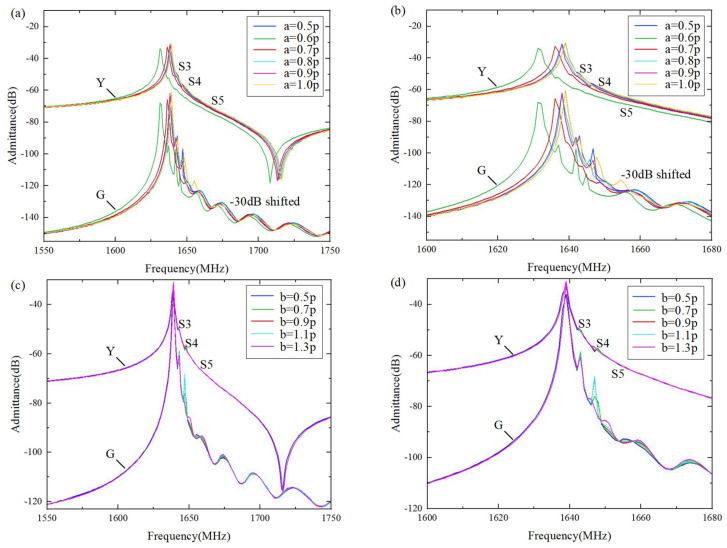
Calculated admittance and conductance with (**a**) dummy widths ranging from 0.5p to 1.0p, and (**c**) dummy lengths ranging from 0.5p to 1.0p. (**b**,**d**) are enlarged views of (**a**,**c**), respectively.

Figure 13a illustrates the effect of the dummy width (ranging from 0.5p to 1.0p (p = 0.5*λ*)) on the suppression of transverse wave modes. It can be seen clearly that configuring the dummy width and length is an efficient way to suppress the transverse wave modes, especially transverse modes S3 and S4. When the metallization ratio of the dummy changed from 0.5p to 1.0p, the amplitude of the transverse modes first decreased and then increased (Figure 13a). The optimal suppression of the transverse modes can be achieved at a = 0.7p. Figure 13b shows the effect of the dummy length, b, (ranging from 0.5p to 1.3p) on the suppression of the transverse wave modes. It is obvious that the dummy length only had a marked impact on the S4 mode, which showed a decreasing trend as the dummy length monotonically increased.

### 3.3. Experimental Verification

**Figure 14:**
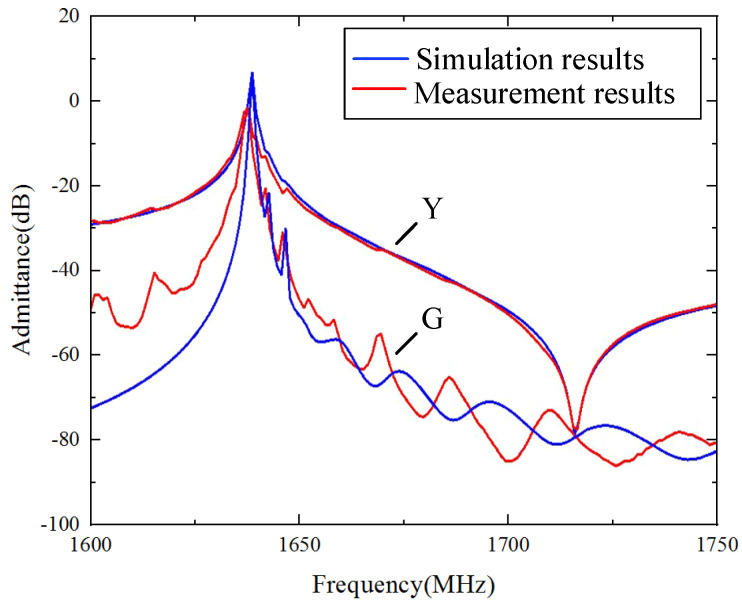
Calculated and measured frequency responses of Al electrode (100 nm)/42°Y-X LT (600 nm)/SiO_2_ (500 nm)/poly-Si (1000 nm)/Si substrate.

A SAW resonator on a multi-layered Al/42°Y-X LT/SiO_2_/poly-Si/Si structure was fabricated, with an Al electrode thickness of 100 nm, a LiTaO_3_ thickness of 600 nm, a SiO_2_ thickness of 500 nm, and a poly-Si thickness of 1000 nm. The IDTs consisted of 151 fingers and 20 fingers of reflectors on both sides. The gap length was 0.35p, the aperture length was 15*λ*, the dummy width was 0.5p (which is equal to the IDT width), and the dummy length was 1.0p. Figure 14 shows the measured (red lines) and calculated (blue lines) admittance Y_11_ curves of the SAW devices. The simulated results are in agreement with the experiment results for the fundamental wave mode. Notably, there was also a weak transverse high-order wave near the main resonant frequency.

**Figure 15:**
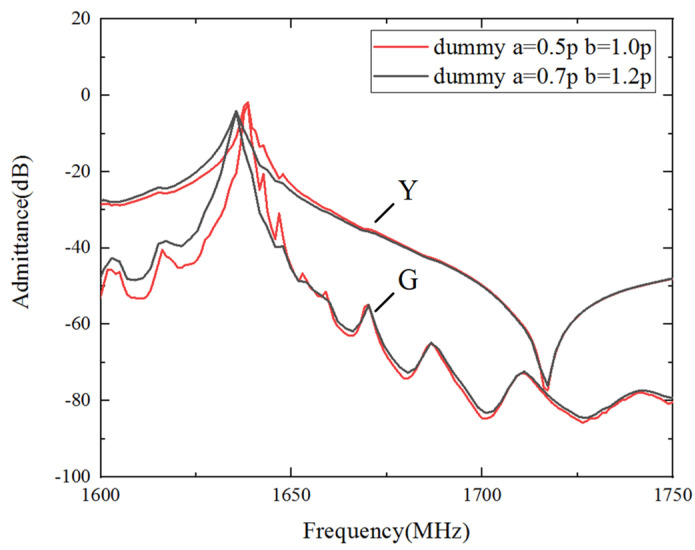
Measured admittance of different dummy structures.

As mentioned above, the unmanageable manufacturing errors probably accidentally yield spurious waves. In order to ensure a good production performance, the dummy structure to improve the SAW performance was also taken into consideration. Thus, a one-port resonator with a dummy width of 0.7p and a dummy length of 1.2p was fabricated. The other parameters remained the same. As shown in Figure 15, the black line shows the frequency response of the re-designed dummy structure, while the red line shows the original dummy structure (a dummy width of 0.5p and a dummy length of 1.0p). The transverse modes near the resonant frequency of the re-designed structure almost completely disappeared.

## 4. Conclusions

In this paper, SAW resonators on an Al/42°Y-X LT/SiO_2_/poly-Si/Si-layered structure were proposed and analyzed. A comprehensive analysis including the *V*_p_, *K*^2^, Bode *Q* value, and transverse wave modes was performed on the proposed SAW resonator full-scaled 3D finite element model. Meanwhile, the dispersion and slowness curves were calculated, which are required to determine if there is good energy confinement and suppression of transverse modes. In addition, the effects of dummy electrodes on transverse waves were discussed. By optimizing its device structure parameters and configuration, an SAW resonator with improved performance and transverse mode suppression was achieved. Furthermore, one-port resonators were fabricated on the optimized Al/42°Y-X LT/SiO_2_/poly-Si/Si-layered structure, and the experimental results were basically consistent with the theoretical calculations. These results give an insight into the general design process for layered SAW devices, which provides guidelines for the design of desired SAW devices with improved performance.

## Figures and Tables

**Table 1:** Material constants used in the calculation.

	Symbol	LiTaO_3_	SiO_2_	Poly-Si	Si
Elastic Constants (×10^10^ N/m^2^)	*C*_11_ *C*_12_ *C*_13_ *C*_33_ *C*_44_	23.29 46.89 80.23 27.53 93.89	7.85 1.61 1.61 7.85 3.12	-	-
Piezoelectric Constants (C/m^2^)	*e*_15_ *e*_31_ *e*_33_	2.59 0.08 1.88	-	-	-
Dielectric Constants	$\varepsilon_{11}/\varepsilon_{0}$ $\varepsilon_{33}/\varepsilon_{0}$	40.9 43.3	3.75 3.75	4.5	11.7
Density (kg/m^2^)	ρ	7450	2200	2320	2329

## Data Availability

The authors have access to all the data in the study (for original research articles) and accept responsibility for their validity.
